# DFSNet: directional feature aggregation and shape-aware supervision for eggplant pest and disease detection

**DOI:** 10.3389/fpls.2026.1775987

**Published:** 2026-02-09

**Authors:** Hui Sun, Weicun Fan, Junbo Zhang, Minghan Feng, Fulin Wang, Rui Fu

**Affiliations:** 1 Weifang University of Science and Technology, Weifang, China; 2 Shandong First Medical University & Shandong Academy of Medical Sciences, Jinan, China

**Keywords:** deep learning, edge feature enhancement, eggplant disease detection, multi-scale attention, real-time detection

## Abstract

In natural planting environments, pest and disease detection on eggplant fruits is characterized by small lesion sizes, weak edge feature information, significant scale variations, and complex backgrounds. Particularly, fruit borer holes, fruit rot lesions, and melon thrips bite marks exhibit obvious differences in size, edge structure, and spatial distribution, posing considerable challenges for real-time accurate detection. This paper proposes the DFSNet, a lightweight improved network for pest and disease detection on eggplant fruits in natural scenes. First, PConv is introduced in the P1, P2 shallow feature extraction stages of the baseline model’s backbone network to enhance the modeling capability for fine-grained directional textures and weak edge information. Subsequently, an MSDA (Multi-Scale Directional Aggregation) module is designed and embedded into the feature enhancement modules at the P3, P4, and P5 layers of the backbone, which effectively improves the perception capability for insect hole edges and lesion contours through multi-directional depthwise separable convolution and Directional Edge Enhancer (DEE). Furthermore, a CSP-MSLA structure is introduced into the neck network, combining multi-scale linear attention mechanism with cross-stage partial connections to achieve selective enhancement of key pest and disease regions while maintaining low computational complexity. Finally, an SDDH (Shape-based Dynamic Detection Head) is introduced, which enhances the model’s adaptive capability to different pest and disease geometric features and scale variations by introducing Scale-based Dynamic Loss. Experimental results demonstrate that the model achieves Precision of 81.0%, Recall of 78.3%, and mAP@50 of 80.5% on a self-constructed eggplant pest and disease dataset under natural scenes, representing improvements of 6.9, 8.8%, and 7.8% percentage points respectively compared to the baseline model. Meanwhile, the model parameters and computational cost are compressed to 1.8M and 5.4G respectively, with an inference speed of up to 378.13 FPS. The proposed method effectively improves small target detection accuracy and robustness under complex backgrounds while ensuring real-time performance, demonstrating particularly significant advantages in detecting small targets such as fruit borer holes and melon thrips bite marks, proving that this model is an efficient and robust real-time detection model for eggplant fruit pests and diseases.

## 1 Introduction

**Figure 1:**
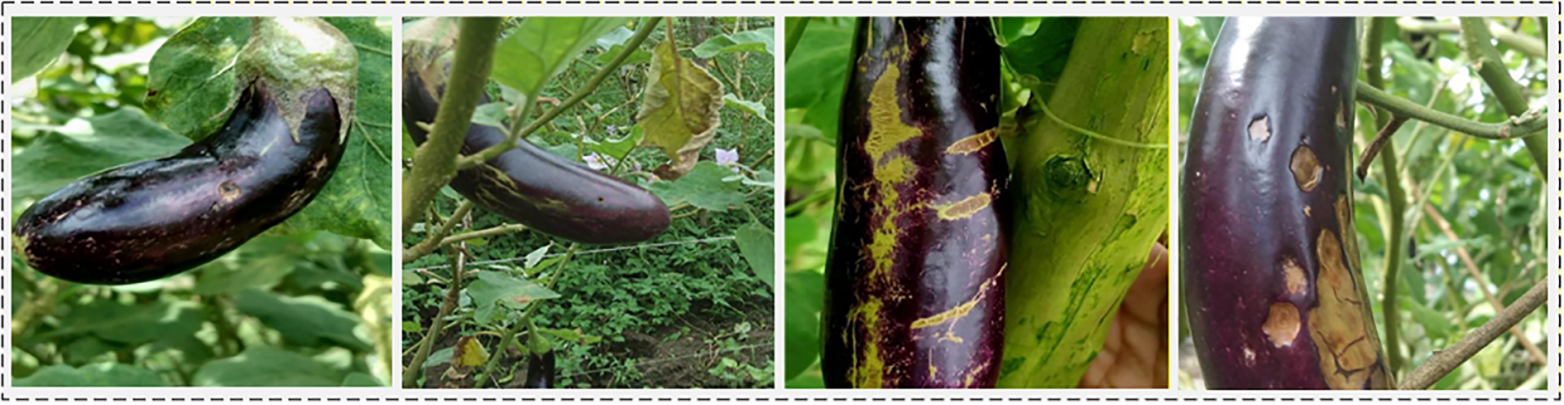
Fruit borer holes (nearly circular cavities) and banded stripes caused by melon thrips feeding, as well as fruit rot caused by fungi, occurring in eggplant cultivation.

Eggplant (Solanum melongena L.), rich in various vitamins and bioactive substances, is one of the widely cultivated vegetables around the world. However, during the growth cycle, fruit diseases become a significant obstacle to eggplant production, such as internal boring caused by fruit borers, fruit rot caused by fungi or bacteria, and surface scars and banded stripes caused by thrips feeding (such as melon thrips), as shown in **Figure 1**. These pests and diseases lead to the loss of fruit commercial value, severely causing enormous economic losses, affecting the economic income of plantations and the production enthusiasm of practitioners (Kellab et al., 2025). Traditional pest and disease diagnosis mainly relies on field inspection and empirical judgment by agricultural experts. This method depends on growers’ visual observation and production experience, with diagnostic results easily influenced by personal experience and conditions. Meanwhile, it is difficult to achieve rapid inspection over large areas and cannot meet the requirements for accurate identification operations in modern large-scale agricultural production. Therefore, developing more efficient and intelligent detection technologies has become an urgent problem to be solved in modern agriculture.

Early research was mostly based on traditional machine learning (Agarwal et al., 2020), performing disease classification through manually designed features, such as lesion segmentation (Spisni et al., 2020) and SVM classification (Wu et al., 2014). However, although such methods have high computational efficiency, their feature representation capability is limited, and generalization performance is poor under complex field conditions such as illumination variations and background interference. In contrast, deep learning methods (Fu et al., 2025) have achieved significant progress in the fields of agricultural monitoring and disease detection, opening up new pathways for intelligent agricultural management. Among them, Convolutional Neural Networks (CNNs) have demonstrated excellent performance in crop disease recognition and classification tasks, with accuracy rates substantially surpassing traditional methods. For example (Ashurov et al., 2025), proposed a DCNN model integrating depthwise separable convolution, SE module, and improved residual connections, achieving a significant reduction in computational complexity and enhancing disease recognition capability in resource-constrained environments with an accuracy of 99.47% (PlantVillage dataset) (Shafik et al., 2025). proposed a plant pest and disease detection method based on ResNet-9 deep convolutional neural network, which not only improved detection accuracy to 97.4%, but also effectively alleviated the class imbalance problem in the dataset through data augmentation strategies (Salka et al., 2025). reviewed and compared CNN-based plant disease detection architectures, establishing EfficientNet-B4 with an accuracy of 99.97% as the current accuracy benchmark. Recent studies have explored target perception and image quality enhancement in complex environments (Li et al., 2025a). introduced a joint detection and tracking framework based on reinforcement learning, which improves the perception of weak targets under heavy clutter (Wang et al., 2023). addressed underwater image degradation through color compensation and multi-attribute adjustment. Later (Wang et al., 2026), proposed a multimodal diffusion model to enhance color fidelity and detail representation under limited data conditions. In addition (Li et al., 2025b), employed graph convolutional networks to exploit echo topology, improving target discrimination in low signal-to-noise scenarios. These studies provide useful insights for perception modeling in challenging environments. Although existing research has made certain progress, disease detection still faces severe challenges in complex agricultural scenarios. As shown in **Figure 1**, the lesions of fruit rot, fruit borer damage, and melon thrips on eggplant fruits are extremely small in size and easily interfered with by complex backgrounds such as leaf textures, illumination shadows, and fruit surfaces, resulting in extremely low pixel proportions of lesion regions and weak visual features. This is because the spatial resolution of single-scale features is insufficient, making it difficult for the model to effectively capture the fine-grained geometric and texture information of small targets, thus causing high miss detection rates and false detection rates, which severely restricts the improvement of detection performance.

To address this, researchers have enhanced the model’s representation capability for targets at different scales by introducing multi-scale feature fusion and attention mechanisms (Zhang et al., 2025a). proposed MAVM-UNet based on multi-scale aggregated vision Mamba, achieving pixel accuracy and MIoU of 82.07% and 81.48% respectively, with performance superior to HCFormer and VM-UNet (Zhang et al., 2023). designed DBCLNet, a dual-branch collaborative network combining multi-scale convolution and Focal Loss, achieving an accuracy of 99.89% on the PlantVillage dataset, significantly surpassing existing mainstream models. Furthermore, WMC-RTDETR proposed by (Zhang et al., 2025b) enhanced multi-scale feature extraction by integrating CSRFPN, achieving 97.7% mAP50 while reducing computational cost by 40.42%, enabling real-time detection on edge devices.

However, although multi-scale feature fusion and attention mechanisms have achieved positive progress, existing methods still have the following limitations: (1) channel and spatial attention based on global pooling are difficult to effectively model cross-scale high-dimensional feature dependencies; (2) complex attention structures are sensitive to noisy features, affecting the robustness of small target detection; (3) the introduction of multi-scale structures often leads to a significant increase in network parameters and computational complexity, which is unfavorable for lightweight deployment and real-time applications.

To address the above problems, this paper proposes a lightweight detection framework integrating edge feature enhancement, multi-scale feature modeling, and efficient feature selection. The main contributions of this study are as follows:

- PConv backbone design for shallow detail perception. To address the problems of blurred edges and weak directional texture features of pest and disease targets, this paper introduces PConv (Pinwheel Convolution) in the shallow stages (P1–P2) of the lightweight detection network. This module models local directional structural information in parallel through multi-directional asymmetric convolution kernels. It significantly enhances the representation capability of direction-sensitive features with minimal parameter increase, effectively improving the feature representation quality of small insect holes and early lesions, and lays a reliable low-level feature foundation for subsequent multi-scale feature fusion.
- MSDA structure for multi-directional multi-scale feature aggregation. To address the limitations of the C3K2 module in directional information modeling, this paper designs the MSDA (Multi-Scale Directional Aggregation) structure and embeds it into C3K2. This module models horizontal and vertical structural information in parallel through multi-directional depthwise separable convolution branches, while introducing a dual-branch edge enhancement module (DEE) to explicitly enhance the edge response of pest and disease targets, thereby improving the model’s recognition capability for fruit rot lesion contours and irregular insect bite marks.
- Lightweight attention-driven CSP-MSLA neck structure. To address the problems of complex background interference and redundant features, this paper constructs the CSP-MSLA structure in the neck network, combining Multi-Scale Linear Attention (MSLA) with the CSP mechanism to achieve adaptive enhancement of key pest and disease regions while controlling computational complexity. This structure improves the discriminability of multi scale feature fusion and enhances the detection robustness of the model under complex illumination variations and local occlusion conditions.
- Shape-aware dynamic detection head SDDH. To address the significant differences in scale distribution and geometric morphology among different pest and disease targets, this paper designs a shape-aware detection head structure and adopts Scale-based Dynamic Loss as the regression supervision strategy. This method alleviates the problem of insufficient gradient contribution of small-scale targets during the training process through a scale-adaptive dynamic supervision mechanism, further improving the generalization capability of the detection head for multi-scale pest and disease targets.

## 2 Related work

### 2.1 Lightweight object detection backbone network structure

In recent years, with the widespread application of object detection algorithms in mobile and embedded scenarios, lightweight network design has become a research hotspot. CNN-based MobileNets (Howard et al., 2019, 2017; Sandler et al., 2018) significantly reduced computational complexity by replacing standard convolutions with depthwise separable convolutions, while GhostNets (Han et al., 2020; Liu et al., 2024; Tang et al., 2022) her reduced the number of parameters by generating feature maps on half of the channels using cheap operations. However, these methods are limited by local receptive fields and struggle to capture global context information. In contrast, Vision Transformer (ViT) demonstrates advantages with its global receptive field and long-range dependency modeling capability. However, the quadratic computational complexity of its self-attention mechanism brings higher computational overhead. To achieve a better trade-off between speed and accuracy, single-stage detection models represented by the YOLO (You Only Look Once) series have achieved a balance between efficiency and performance in real-time object detection tasks through collaborative optimization design of the backbone network, neck structure, and detection head.

Among them (Pan et al., 2025), constructed the SSD-YOLO model by integrating the SENetV2 mechanism and DySample lightweight sampling module, achieving efficient and accurate detection of rice diseases with only 6MB parameters (Song et al., 2024). constructed the extremely lightweight model DODN by fusing deformable convolution and Transformer components, achieving efficient and accurate detection of cucumber diseases in complex scenarios with only 3.7 MB parameter scale and 3.9 GFLOPs low power consumption. However, such methods rely on spatial convolution stacking, which is not only limited by computational resources, but also difficult to adapt to pest and disease detection due to the neglect of fine-grained textures, resulting in existing models often being in a suboptimal state in real agricultural scenarios, which is also the core problem that this paper urgently needs to solve.

### 2.2 Frequency domain feature modeling and the application of wavelet transform in visual tasks

In addition to traditional spatial domain convolution, frequency domain feature modeling has gradually received attention in recent years. MWCNN proposed by (Liu et al., 2018) introduced Discrete Wavelet Transform (DWT) to replace traditional downsampling, retaining frequency domain information while compressing feature maps, effectively alleviating the information loss problem (Li et al., 2020). achieved decoupling of high-frequency and low-frequency components through DWT, significantly enhancing the anti-noise robustness of the model. Wavelet-SRNet proposed by (Huang et al., 2017) utilized wavelet coefficient prediction to reconstruct facial details, solving the over-smoothing problem in super-resolution tasks. Wavelet transform, with its excellent time-frequency localization characteristics, achieves effective decoupling of structure and details through multi-scale frequency band decomposition, demonstrating significant advantages in tasks such as image restoration, super-resolution, and semantic segmentation.

In the field of pest and disease detection, existing lightweight models neglect fine-grained textures due to their reliance on spatial convolution stacking, making it difficult to cope with complex detection scenarios. To address this, researchers have attempted to introduce wavelet transform to enhance texture perception capability (Li et al., 2022). introduced Discrete Wavelet Transform (DWT) into YOLOv4, strengthening the extraction of pest and disease detail textures and achieving accurate detection of small targets under complex backgrounds (Li et al., 2022). utilized Continuous Wavelet Analysis (CWA) to process hyperspectral data, accurately discriminating the stress states of tea plants affected by tea green leafhoppers, anthracnose, and other similar symptoms (Panchananam et al., 2025). proposed WFS-YOLO, which enhanced features in both frequency domain and spatial domain through Discrete Wavelet Transform (DWT), improving the perception accuracy of small pests and diseases in complex environments.

However, such methods are difficult to adapt to lightweight deployment due to their structural complexity and computational expense. How to efficiently utilize frequency domain information under conditions of limited computing power remains a core challenge in current model design.

### 2.3 Edge and high-frequency feature enhancement methods

Edge information is an important basis for target contour and shape discrimination. In fine-grained visual tasks such as pest and disease detection, high-frequency textures and boundary features are crucial for distinguishing lesions, insect holes, and healthy regions. However, low-resolution images lose a large amount of high-frequency details during the imaging process, resulting in edge blurring and texture degradation. To solve this problem, researchers have explored enhancement strategies for edge and high-frequency features from multiple perspectives. For example (Zhao et al., 2019), proposed EGNet, which guides target localization by explicitly modeling edge features (Edge Guidance Stream), compensating for the loss of boundary information in deep networks (Qiu et al., 2024). proposed an Adaptive Compressed Sensing (ACS) architecture that captures key edge regions through a cascaded guidance mechanism, providing a low-overhead solution for pest and disease detail preservation (Zheng and Yang, 2024). proposed Contextual Boundary Aware Network (CBA-Net), which strengthens the model’s capture of salient object contours through a contextual boundary awareness mechanism.

Although existing methods have made progress in edge and high-frequency feature enhancement, they still have limitations in fine-grained tasks such as pest and disease detection. Existing methods mostly focus on salient edges, with insufficient reconstruction capability for small textures such as early lesions and insect holes; frequency domain and spatial domain mechanisms are often designed independently, making it difficult to collaboratively capture global and local features; in addition, improper module design can easily lead to a surge in computational overhead or feature distribution imbalance. Therefore, how to design a lightweight and efficient enhancement mechanism that balances fine-grained texture recovery and global reconstruction quality is the core problem of this paper.

### 2.4 Application of multi-scale attention mechanisms

Multi-scale feature fusion is a key technology for improving object detection performance. Classic structures such as FPN and PAN fuse features at different scales through top-down and bottom-up pathways, but their information interaction mainly relies on element-wise addition or concatenation, lacking explicit modeling of cross-scale semantic relationships. In recent years, attention mechanisms have been introduced into detection networks to enhance feature selectivity and context awareness capability. For example (Sun et al., 2025), proposed the SRCA attention module, which effectively integrates high and low-resolution features through adaptive weighting and bidirectional fusion, significantly improving the multi-scale perception capability of tomato leaf lesions (Zhang, 2025). designed the MHCF encoder, which enhances multi-scale feature fusion using the Transformer structure, achieving a balance between accuracy and efficiency in pomegranate detection in complex orchard environments.

However, existing attention-based multi-scale fusion methods are usually accompanied by high computational complexity, making it difficult to directly adapt to resource-constrained lightweight detection models. How to design computationally efficient attention mechanisms while maintaining the effectiveness of multi-scale feature fusion, achieving a balance between detection accuracy and model lightweight, is the core problem that this paper is committed to solving.

## 3 Methods

### 3.1 Overall network architecture

**Figure 2:**
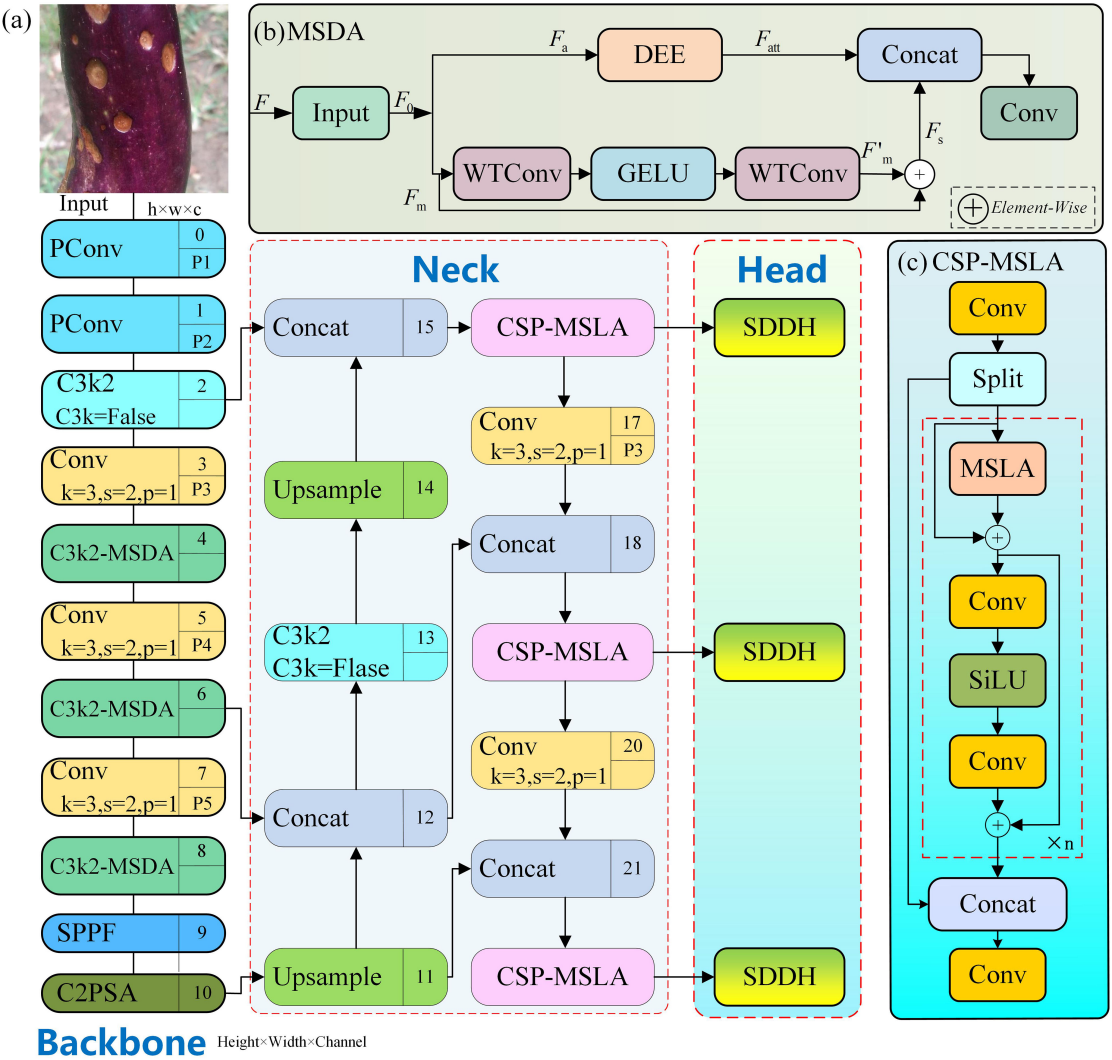
Overall architecture of DFSNet: **(a)** the complete network,**(b)** the MSDA model and **(c)** the CSP-MSLA model.

Based on the baseline model lightweight detection framework, this paper constructs an improved model DFSNet (Directional Feature Aggregation and Shape-Aware Supervision for Eggplant Pest and Disease Detection) for pest and disease detection on eggplant fruits in natural scenes. The structure is shown in **Figure 2a**. While maintaining the original inference efficiency advantages, DFSNet performs targeted optimization on backbone feature extraction, feature fusion, and detection head, specifically addressing the characteristics of pest and disease targets such as “small scale, multiple morphologies, and weak edges”.

In the backbone network, Conv is replaced by PConv (Pinwheel Convolution) in the P1–P2 layers to enhance shallow directional texture and fine-grained edge feature representation; meanwhile, the MSDA (Multi-Scale Directional Aggregation, as shown the **Figure 2b** module is introduced into the C3K2 structure to improve the aggregation capability for multi-directional structural information. In the neck network, the CSP-MSLA structure (as shown in the **Figure 2c** is designed to integrate the multi-scale linear attention mechanism into the cross-stage partial connection framework to achieve selective enhancement of key pest and disease regions. Finally, SDDH (Shape-Based Dynamic Detection Head) is introduced to improve the model’s adaptive capability to different pest and disease geometric features through a shape aware dynamic loss function.

### 3.2 PConv-enhanced shallow feature extraction

**Figure 3:**
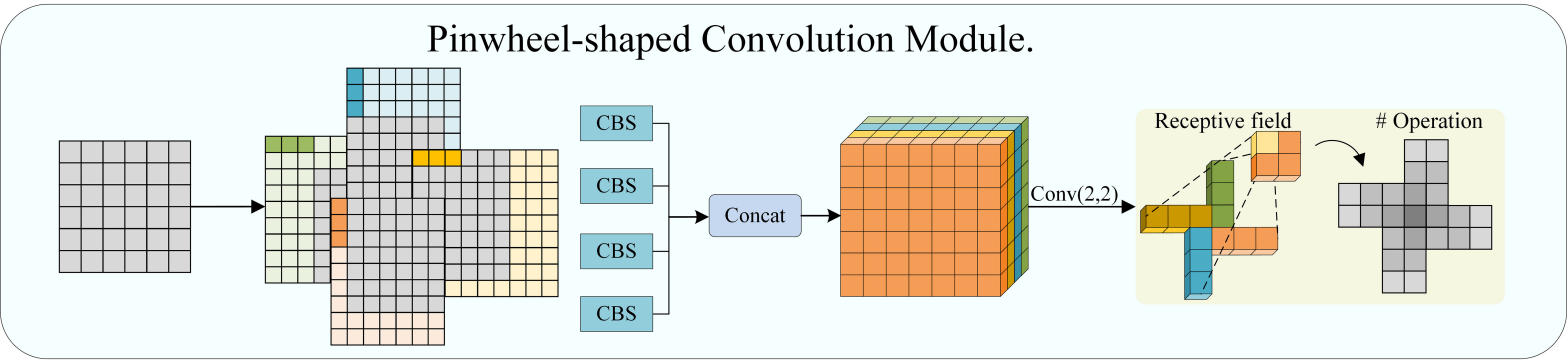
Pinwheel-shaped convolution module. The CBS module consists of three parts: Conv, BN (Batch Normalization), and SiLU. Concat stands for concatenate.

In the baseline model YOLOv11 original network, backbone feature extraction mainly relies on standard two-dimensional convolution operators. The convolution kernels in standard convolution have a unified response form in all spatial directions. This modeling approach implicitly assumes that local structures have similar statistical characteristics in different directions. However, in natural scene eggplant fruit pest and disease detection, this assumption is difficult to establish. For example, fruit borer holes typically exhibit extremely small scale and weak edge local structures, while melon thrips bite marks present obvious elongated stripe morphology with strong directional dependence. Standard convolution has limited discriminative capability for these directional features in the shallow stages, easily leading to the weakening of key information during the feature downsampling process. Therefore, this paper introduces the PConv (Pinwheel Convolution) structure in the P1, P2 layers of the YOLOv11 backbone network, as shown in **Figure 3**, to replace standard convolution.

PConv (Yang et al., 2025) achieves explicit modeling of local structural directionality by introducing asymmetric padding strategies in different spatial directions of the input feature map and combining parallel convolution operations. Specifically, for the four directions of left, right, top, and bottom, different forms of asymmetric padding are applied respectively, as shown in Equation 1:

(1) $$X^{\left(d\right)}=P^{\left(d\right)}\left(X\right),\;d\in \left\{\text{left},\text{right},\text{top},\text{bottom}\right\}$$

where $P^{\left(d\right)}$ represents the asymmetric padding operation applied along the *d*-th direction, used to introduce directionally biased spatial context information. Subsequently, CBS operations are performed on the four directionally enhanced feature maps respectively, with the expression given by Equation 2:

(2) $$X_{i}=\text{SiLU}\left(\text{BN}\left(X^{\left(d\right)}\right)\;\text{one}\;k_{i}\right),\;i\in \left\{1,2,3,4\right\}$$

where *k_i_*is the convolution kernel. Finally, the output feature *X*_out_ is obtained, as shown in Equation 3:

(3) $$X_{out}=\text{SiLU}\left(\text{BN}\left(\text{Concat}\left(X_{1},X_{2},X_{3},X_{4}\right)\right)\text{ one }K_{2\times 2}\right)$$

Compared to traditional convolution, this approach can more sensitively capture target structures with obvious directional features, such as lesion edge contours and elongated insect bodies, while maintaining a low number of parameters. By introducing PConv before attention modeling, the model can proactively highlight morphological information related to pests and diseases and suppress the interference of complex background textures on subsequent feature fusion processes. Meanwhile, explicit directional structural modeling capability is introduced at the shallow stage, enhancing the discriminability of edge and texture features while maintaining lightweight characteristics.

### 3.3 C3K2-MSDA

**Figure 4:**
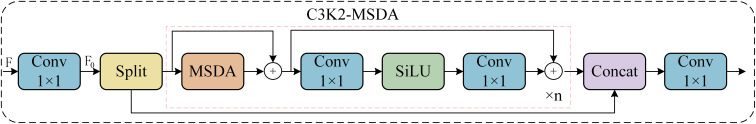
Architecture of C3K2-MSDA.

Although introducing directional convolution PConv at shallow layers can enhance detail representation, the baseline model adopting small convolution kernels and shallow design is prone to insufficient context modeling and insufficient receptive field problems when dealing with large targets and complex backgrounds. This paper introduces a wavelet-enhanced multi-scale dilated attention module MSDA (Multi-Scale Directional Aggregation) into C3K2 at the P3, P4, and P5 stages of the backbone, obtaining C3K2-MSDA as shown in **Figure 4**. In this module, one path maintains the original cross-stage connection path of C3K2, while the other path introduces MSDA for enhanced modeling, with the structure shown in **Figure 2b**-MSDA. This design ensures the continuity of the original C3K2 feature flow while providing additional multi-scale attention supplementation to the network. The MSDA structure consists of two important branches: multi-scale modeling and EdgeEnhance branch. Let the input feature be $F\in {\mathbb{R}}^{C\times H\times W}$, where C, H, and W represent the number of channels, height, and width respectively.

First, a 1×1 convolution is used to complete channel mapping as shown in Equation 4:

(4) $$F_{0}={\text{conv}}_{1\times 1}\left(F\right)$$

#### 3.3.1 Multi-scale modeling branch

The multi-scale modeling branch adopts a continuous WTConv structure combined with the GELU nonlinear function as shown in **Figure 2b**, and the feature transformation process can be expressed as Equation 5:

(5) $$F_{0}=\left[F_{m},F_{a}\right]$$

where *F_m_*is used for multi-scale feature modeling, and *F_a_*is used for attention weight generation. In the multi-scale branch, a continuous feature transformation structure based on WTConv is introduced to enhance the perception capability for local patterns at different scales. The feature extraction process of this branch can be expressed as Equation 6:

(6) $$F{'}_{m}=\phi_{w_{2}}\left(\delta \left(\phi_{w_{1}}\left(F_{m}\right)\right)\right)$$

where $\phi_{w_{1}}\left(\cdot \right)$ and $\phi_{w_{2}}\left(\cdot \right)$ represent WTConv operations, and δ(·) is the GELU nonlinear activation function. To avoid information attenuation in deep networks while enhancing feature stability, the feature $F_{ms}$ after introducing the residual connection is shown in Equation 7:

(7) $$F_{ms}=F_{m}+F{'}_{m}$$

**Figure 5:**
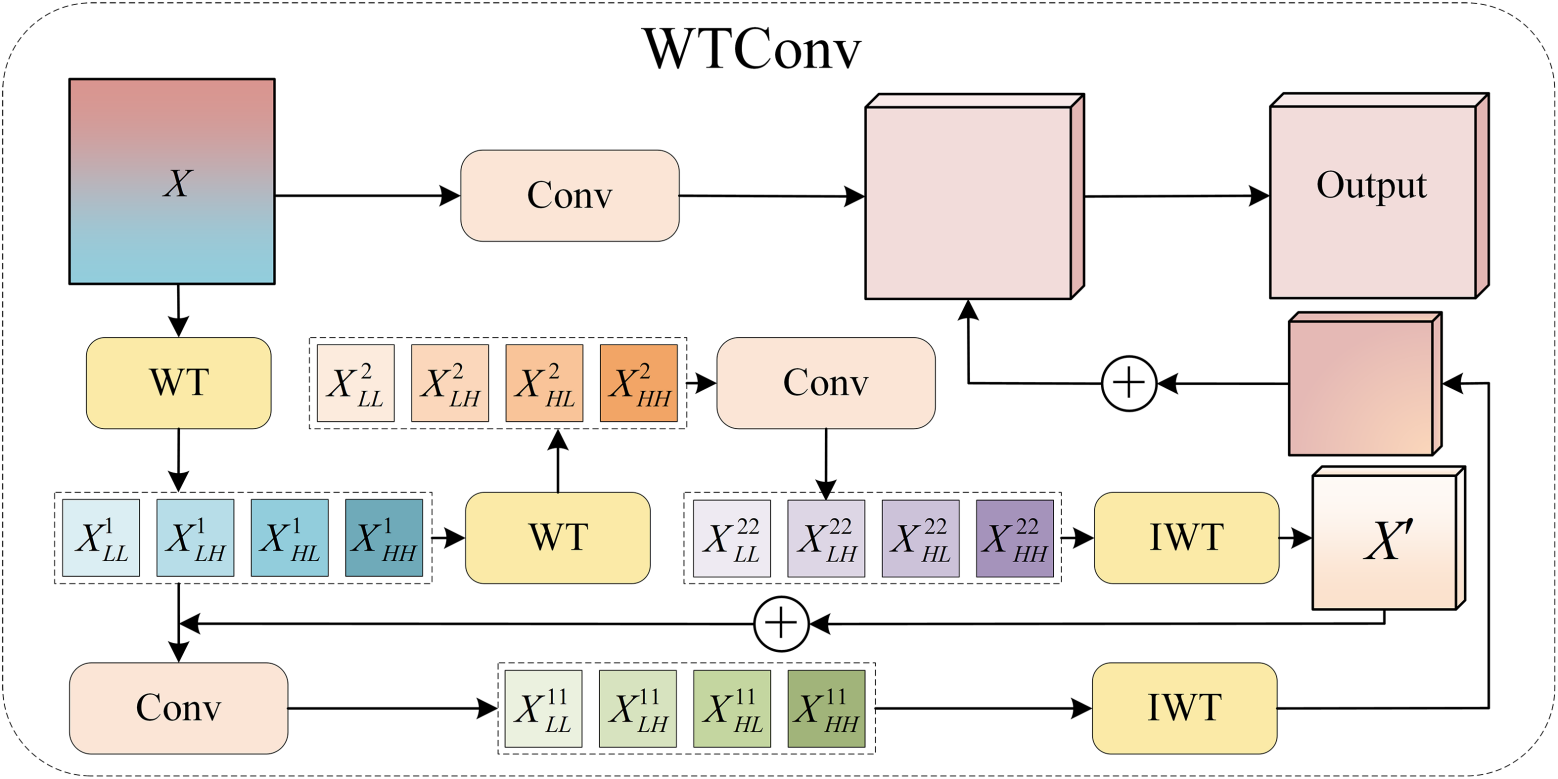
Architecture of WTConv.

This structure enables multi-scale features to obtain richer contextual representations while maintaining original structural information. WTConv (Finder et al., 2024) (as shown in **Figure 5**), as a key component for implementing large receptive field modeling in the MSDA module, is used to enhance the global representation capability of features while maintaining local structural information. WTConv decomposes the input feature map into four frequency subbands through discrete wavelet transform, including LL, LH, HL, and HH. Among them, LL mainly reflects the overall structure and semantic information of the target, while (LH, HL, and HH) correspond to detail features such as edges and textures. By independently modeling features in different frequency bands and fusing them in subsequent stages, WTConv can introduce cross-scale and cross-frequency feature responses without significantly increasing computational complexity. This multi-band feature aggregation approach enables the network to simultaneously perceive local details and larger-scale contextual information, thereby effectively expanding the receptive field in the MSDA module and enhancing the representation capability for edges and structural changes of pest and disease targets.

#### 3.3.2 Edge feature enhancement module DEE

**Figure 6:**
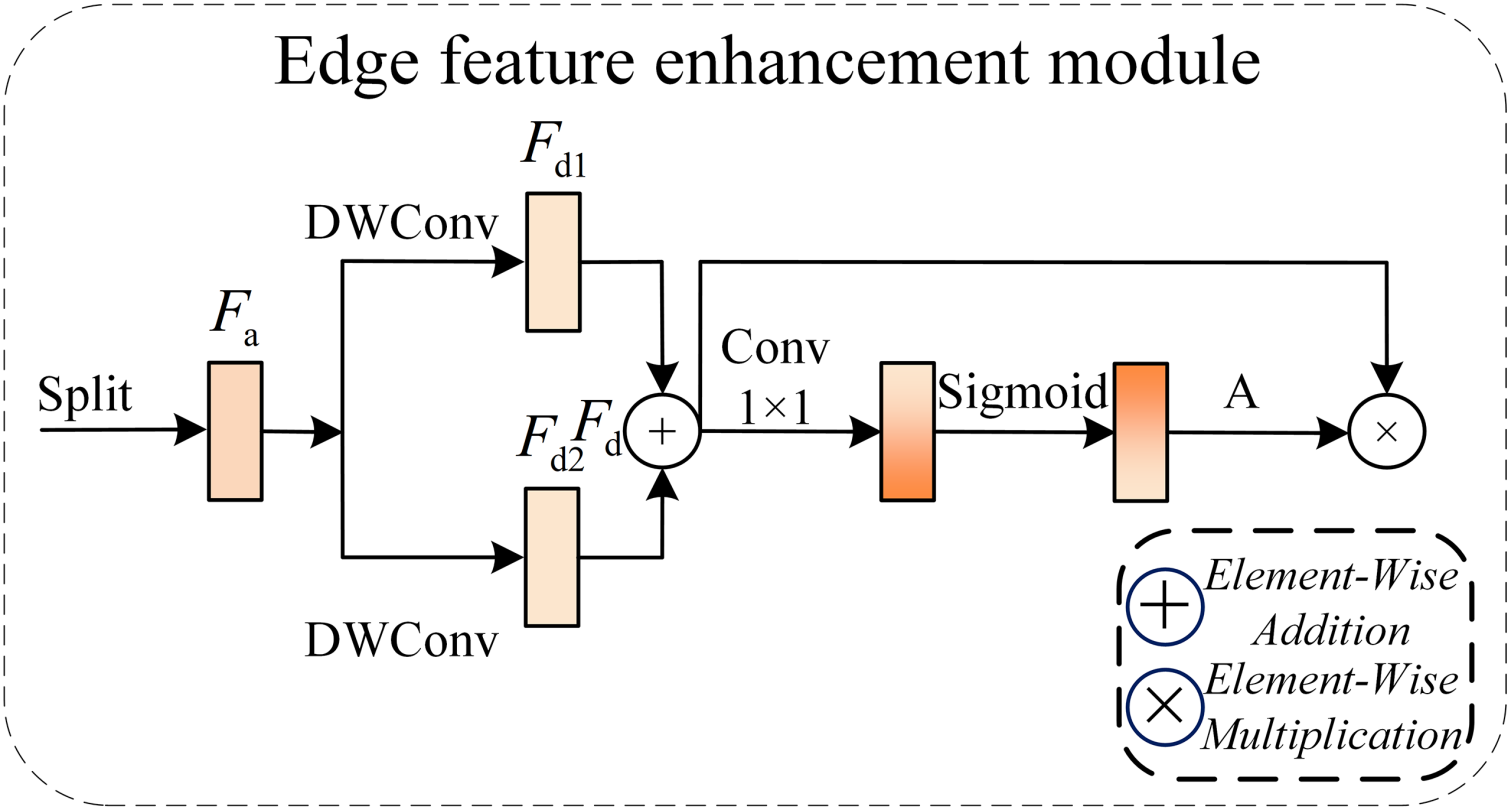
Architecture of DEE module.

In pest and disease detection tasks, the edges of pests and diseases such as fruit borer holes, fruit rot lesions, and melon thrips stripes present rapidly changing pixel intensity regions on eggplant fruit surface images. These regions change dramatically and constitute high-frequency information, which is also the core feature for distinguishing pest and disease features from healthy fruit images. Therefore, effective modeling of high frequency edge features can effectively highlight the contour and shape features of pests and diseases. However, traditional convolutional neural networks tend to produce smoothing effects on high-frequency details during layer-by-layer downsampling and feature fusion processes, resulting in the gradual weakening of edge information. To address this problem, this paper designs DEE (Edge feature Enhancement Module) as shown in **Figure 6**, which explicitly highlights high-frequency change regions in the input features, enabling the network to perceive the contour and shape information of pest and disease regions during the feature extraction stage.

This module models gradient changes in the feature map and injects the enhanced edge response into the original features in residual form, thereby avoiding interference with the overall semantic structure. It is used to effectively strengthen the representation capability of edge and high-frequency information without changing the spatial resolution of the input feature map. The DEE module acts on intermediate layer features of the network, and its enhancement process is learnable, capable of adaptively adjusting the response intensity to different edge patterns according to task requirements.

This module takes intermediate layer feature mapping as input and first calculates feature gradients (F_d1, F_d2) in the horizontal and vertical directions respectively, representing regions with relatively drastic pixel intensity changes in the feature map, thereby explicitly extracting high-frequency information such as edges and textures. Subsequently, a comprehensive edge response is obtained through gradient magnitude fusion, and 1×1 convolution is used for channel mapping and adaptive reweighting of edge features. Finally, the enhanced edge features are combined with the original features in residual form, achieving effective enhancement of target boundaries and local structures while avoiding disruption of the original semantic information distribution. Equations 8-12 express the feature information flow process.

(8) $$F_{d1}=\phi_{w_{1}}\left(F_{a1}\right)$$

(9) $$F_{d2}=\phi_{w_{2}}\left(F_{a2}\right)$$

where $\phi_{dw}\left(\cdot \right)$ represents Depthwise Convolution, used to obtain spatial structural information at lower computational complexity.

(10) $$F_{d}=F_{d_{1}}+F_{d_{2}}$$

Subsequently, 1×1 convolution and Sigmoid function are used to generate attention weight A as shown in the equation:

(11) $$A=\sigma \left(\phi_{1\times 1}\left(F_{d}\right)\right)$$

and perform element-wise recalibration on the input features:

(12) $$F_{att}=F_{d}⨀A$$

where ⊙ represents element-wise multiplication. This process achieves joint feature selection at both spatial and channel levels without introducing global pooling or high-complexity operators.

DEE enhances the activation intensity of pest and disease target edge regions through adaptive weighting of multi-directional feature responses, improving the model’s perception capability for fruit borer hole edges and fruit rot lesion contours. This module maintains a lightweight design 345 in structure, effectively balancing directional information modeling capability and computational 346 efficiency.

### 3.4 CSP-MSLA neck and shape-aware detection head

#### 3.4.1 CSP-MSLA neck design

**Figure 7:**
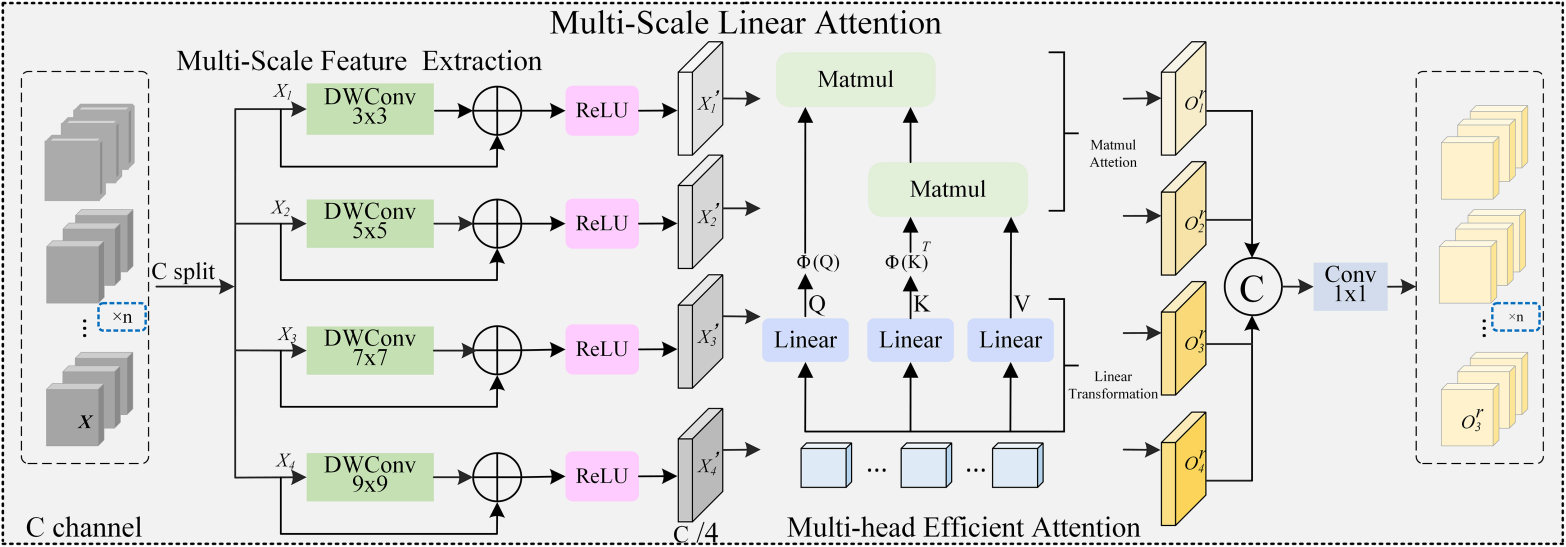
Architecture of MSLA.

In natural scenes, complex illumination, leaf occlusion, and background texture similarity easily introduce a large amount of redundant features, weakening the multi-scale feature fusion effect. In object detection, the Neck structure plays an important role in connecting the backbone network and detection head, with its core objective being to achieve effective alignment and fusion of multi-scale features. The original Neck of YOLOv11n mainly relies on lightweight convolution modules such as C3K2 for feature transformation, possessing certain local modeling capability while ensuring computational efficiency. However, such structures are still essentially dominated by spatial domain convolution, with limited modeling capability for cross-scale contextual relationships and long-range dependencies. Especially in complex agricultural scenarios, the semantic associations between small scale lesions and medium-scale fruit regions are difficult to fully characterize. On the other hand, attention mechanisms demonstrate obvious advantages in modeling long-distance dependencies and global information interaction, but directly introducing standard self-attention structures often brings high computational and storage overhead, making them unsuitable for lightweight detection frameworks. Based on this, in the baseline Neck part, this paper introduces the Multi-Scale Linear Attention (MSLA) module for key scale feature layers P3, P4, and P5, and deeply integrates it with the C3K2 structure to reconstruct the module into CSP-MSLA units as shown in CSP-MSLA in **Figure 2c**. The CSP structure reduces redundant computation and enhances gradient flow through cross-stage feature splitting and recombination. The MSLA structure, as shown in **Figure 7**, explicitly introduces multi-scale global modeling capability, modeling cross-position and cross-scale global association relationships in multi-scale feature space. The combination of the two enables the neck network to significantly enhance the model’s capability to localize pest and disease target regions of interest and suppress irrelevant information while maintaining lightweight characteristics.

In the MSLA (Multi-Scale Linear Attention) module, the three branches Q, K, and V are retained, but to reduce computational complexity, the global computation of QKT is approximated linearly. Meanwhile, multi-scale convolutions (e.g., 3 × 3, 5 × 5, 7 × 7, 9 × 9) are applied to enhance the features of Q and K, and matrix multiplication is used to calculate weights for local regions. That is,


$$Y=\phi \left(Q\right)\cdot (\phi \left(K{)}^{T}\cdot V\right),$$

where ϕ represents a kernel function approximation or multi-scale feature transformation. This transformation bypasses explicit Softmax, achieving attention intensity distribution through kernel function approximation or weight normalization. Due to the adoption of block computation (i.e., first calculating *K^T^V*, then multiplying with *Q*), the overall computational complexity is reduced from *O*(*N*^2^) in traditional self-attention to *O*(*N*). By constructing multi-scale parallel convolution branches, MSLA can capture feature responses under different receptive fields and utilize the linear attention mechanism to perform weighted fusion of multi-scale features, enabling coordinated representation of local detail information and global semantic information. This design promotes effective transfer of multi-scale features in the backbone network and helps improve cross-scale feature modeling capability.

#### 3.4.2 Shape-aware dynamic detection head

##### 3.4.2.1 Baseline model loss function

The baseline model adopts a fixed loss function in the detection head, which consists of the classification loss *L*_cls_, the objectness confidence loss *L*_obj_, and the bounding box regression loss *L*_reg_. The overall formulation can be expressed as Equation 13.

(13) $$L_{\text{YOLOv}11}=L_{\text{cls}}+L_{\text{obj}}+\lambda L_{\text{reg}}$$

Here, *L*_reg_ is typically optimized based on IoU or its variants, and *λ* denotes the weighting coefficient of the regression loss term *L*_reg_. This loss function adopts a unified weight allocation strategy for all targets, without explicitly distinguishing the contributions of objects with different scales or shapes during training. In pest and disease detection tasks, such approximately uniform supervision is prone to causing gradient imbalance. On the one hand, small-scale targets (e.g., borer holes) occupy a relatively small proportion of pixels in the feature maps, making their *L*_reg_ easily overwhelmed in the overall loss. On the other hand, elongated and stripe-like targets (e.g., feeding traces of thrips) are highly sensitive to slight localization deviations under IoU-based constraints, which leads to instability in the regression process. To address these issues, this paper introduces a Scale-based Dynamic Loss on the basis of the original regression structure to dynamically adjust the regression supervision. This strategy is applied to the detection head, forming the SDDH (Shape-aware Dynamic Detection Head).

##### 3.4.2.2 Scale-based dynamic loss

It is well known that IoU-based losses (*S*_loss_) exhibit relatively large fluctuations in small object detection, which negatively affect model stability and regression performance. In *S*_loss_ with bounding box (BBox) annotations, smaller objects usually receive lower attention weights, whereas mask annotations have a greater impact on small or irregularly shaped objects. Therefore, some studies dynamically adjust the influence coefficients *β* of *S*_loss_ and *L*_loss_ according to object scale, so as to enhance the influence of *S*_loss_ on mask annotations and reduce the adverse effects of inaccurate annotations on the stability of the loss function, thereby ensuring that the model pays more attention to small or irregularly shaped objects. This loss function mainly consists of two components: LSDB (the Scale-based Dynamic Loss for the BBox) and LSDM (the Scale-based Dynamic Loss for the Mask). The computation of LSDB and its related parameters are given in Equations 14-20, while the computation of LSDM and its related parameters are presented in Equations 21-23.

• The ${ℒ}_{\text{SDB}}$

The scale-based dynamic loss for the bounding box is composed of a scale consistency loss ${ℒ}_{\text{BS}}$ and a localization loss ${ℒ}_{\text{BL}}$ with corresponding weights. It is defined as Equation 14:

(14) $${ℒ}_{\text{SDB}}=\beta_{1}\times {ℒ}_{\text{BS}}+\beta_{2}\times {ℒ}_{\text{BL}}$$

Here, $\beta_{1}\in \left[0.5,1.0\right]$ and $\beta_{2}\in \left[1.0,1.5\right]$as defined in Equation 18, denote the dynamic weighting coefficients for the bounding box scale loss ${ℒ}_{\text{BS}}$ (see Equation 15) and the localization loss ${ℒ}_{\text{BL}}$ (see Equation 16), respectively. The scale loss is defined as:

(15) $${ℒ}_{\text{BS}}=1-S_{\text{IoU}}+\gamma$$

(16) $${ℒ}_{\text{BL}}=\frac{d^{2}\;\mathrm{one}\;\left(\left(x_{\text{sbp}},y_{\text{sbp}}\right),\left(x_{\text{sgt}},y_{\text{sgt}}\right)\right)}{L^{2}}$$

Let *B*_P_ and *B*_gt_ denote the predicted bounding box and the ground-truth bounding box, respectively. The scale-aware *S*_IoU_ is defined as Equation 17:

(17) $$S_{\text{IoU}}=\frac{B_{\text{P}}\cap \;\mathrm{one}B_{\text{gt}}}{B_{\text{P}}\cup \;\mathrm{one}B_{\text{gt}}}$$

*β*_1_ and *β*_2_ is defined in Equation 18. These coefficients dynamically adjust the loss weights according to the object scale. Here a scale influence factor *β*_3_ is introduced as shown in Equation 19:

(18) $$\beta_{1}=1-\delta +\beta_{3},\;\beta_{2}=1+\delta -\beta_{3}$$

(19) $$\beta_{3}=\text{min }(\frac{B_{\text{gt}}}{\max\;B_{\text{gt}}}\times \theta \times \delta ,\;\delta )$$

where δ =0.5 is the upper limit for scale adjustment, used to constrain the range of weight variation and prevent instability during training, red $\text{max }B_{\text{gt}}=81$ is the maximum size of IRST as defined by the Society of Photo-Optical Instrumentation Engineers (Zhang et al., 2003). The scale mapping factor *θ* is defined as Equation 20:

(20) $$\theta =\frac{{\text{size}}_{i}}{{\text{size}}_{f}}$$

Here, $\beta_{3}$ is the scale influence factor for both the BBox and Mask branches; the function d(·) denotes the Euclidean distance; L represents the diagonal length of the minimum enclosing rectangle that simultaneously bounds the predicted box $B_{\text{P}}$ and the ground-truth box $B_{\text{G}}$, used to normalize the center point distance. ${\text{size}}_{i}$ and ${\text{size}}_{f}$ denote the dimensions of the original image and the current feature map, respectively.

• The ${ℒ}_{\text{SDM}}$

The ${ℒ}_{\text{SDM}}$ is similarly composed of the mask scale loss ${ℒ}_{\text{MS}}$ and the mask localization loss ${ℒ}_{\text{ML}}$ with corresponding weights $\left(\beta_{1}^{'}\in \left[1.0,1.5\right],\beta_{2}^{'}ın\left[0.5,1.0\right]\right)$, as defined in Equation 21:

(21) $${ℒ}_{\text{SDM}}=\beta_{1}^{'}\times {ℒ}_{\text{MS}}+\beta_{2}^{'}\times {ℒ}_{\text{ML}}$$

Let *M*_P_ and *M*_gt_ denote the sets of pixels in the predicted mask and the ground-truth mask, respectively, and let *p* be a weighting coefficient. The mask scale loss ${ℒ}_{\text{MS}}$is defined as Equations 22, 23:

(22) $$M_{\text{IoU}}=\frac{M_{\text{P}}\cap \;\mathrm{one}M_{\text{gt}}}{M_{\text{P}}\cup \;\mathrm{one}M_{\text{gt}}}$$

(23) $${ℒ}_{\text{MS}}=1-p\cdot M_{\text{IoU}}$$

The mask localization loss ${ℒ}_{\text{ML}}$ is defined as Equation 24:

(24) $${ℒ}_{\text{ML}}=1-\frac{\min\;\mathrm{one}\;(d_{\text{mp}},d_{\text{mgt}})}{\max\;\mathrm{one}\;(d_{\text{mp}},d_{\text{mgt}})}+\frac{4\left(\theta_{\text{mp}}-\theta_{\text{mgt}}\right)}{\pi^{2}}$$

Here, *d*_mp_ and *d*_mgt_ denote the average distances of the predicted mask pixels and the ground-truth mask pixels from the origin in polar coordinates, respectively; *θ*_pp_ and *θ*_pgt_ represent the average angles of the predicted mask pixels and the ground-truth mask pixels in polar coordinates, respectively.

The Scale-based Dynamic Loss (SDLoss) employed in this study incorporates object scale information into the loss computation process to achieve adaptive constraints for targets of different scales. In the bounding box regression branch, the scale consistency term and the localization term are combined with weighted summation, and the weights are adjusted using scale factors, thereby applying differentiated supervision to targets of varying scales without altering the original regression formulation. In the mask branch, SDLoss integrates pixel-level overlap constraints with polar-coordinate-based spatial distribution modeling, providing joint constraints on the regional consistency and spatial distribution of mask predictions, which helps improve the modeling stability for irregularly shaped targets. It should be noted that the mathematical definition of the Scale-based Dynamic Loss is not modified in this work; rather, it is applied to pest and disease detection tasks and combined with the proposed detection head structure to accommodate the variations in scale and shape of pest and disease targets in natural scenes.

## 4 Experiments and results

### 4.1 Dataset construction

**Figure 8:**
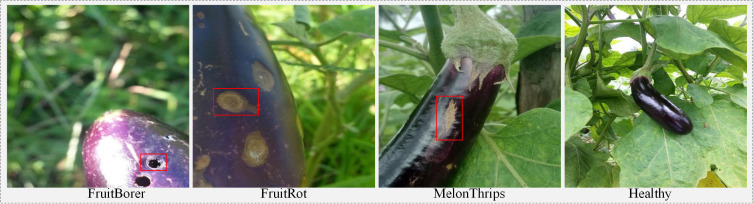
Some samples of eggplant fruit disease dataset.

In this study, a custom dataset of eggplant fruit pests and diseases was established, comprising four categories: FruitBorer, FruitRot, and MelonThrips, as shown in **Figure 8**. The dataset was primarily derived from two sources. The first source consists of sample images collected on October 3, 2025, in a vegetable greenhouse in Shouguang, Shandong Province, China. The original images, captured using an iPhone 14 at a resolution of 1920×1080, encompassed various lighting conditions and shooting angles to enhance data diversity, resulting in 1074 images in JPEG format. After removing blurred, duplicate, and invalid images, 673 valid samples remained. These images were annotated using the Label Studio tool to label the pest and disease regions and their corresponding categories, and the annotations were saved in YOLO format. The second source is the publicly available Eggplant Fruit Disease dataset from the Robotflow platform, from which 2,177 pest and disease images were randomly selected. Combined with the first source, a total of 3,250 sample images were obtained, and all images were resized to 640×640 pixels. To improve model generalization and robustness, Mosaic data augmentation techniques, including random translation, horizontal/vertical flipping, non-uniform scaling, brightness adjustment, and Gaussian noise injection, were applied to expand the number of training images to 7,256. The dataset was randomly split into training (5,080 images), validation (1,451 images), and test sets (725 images) in a 7:2:1 ratio.

**Figure 9:**
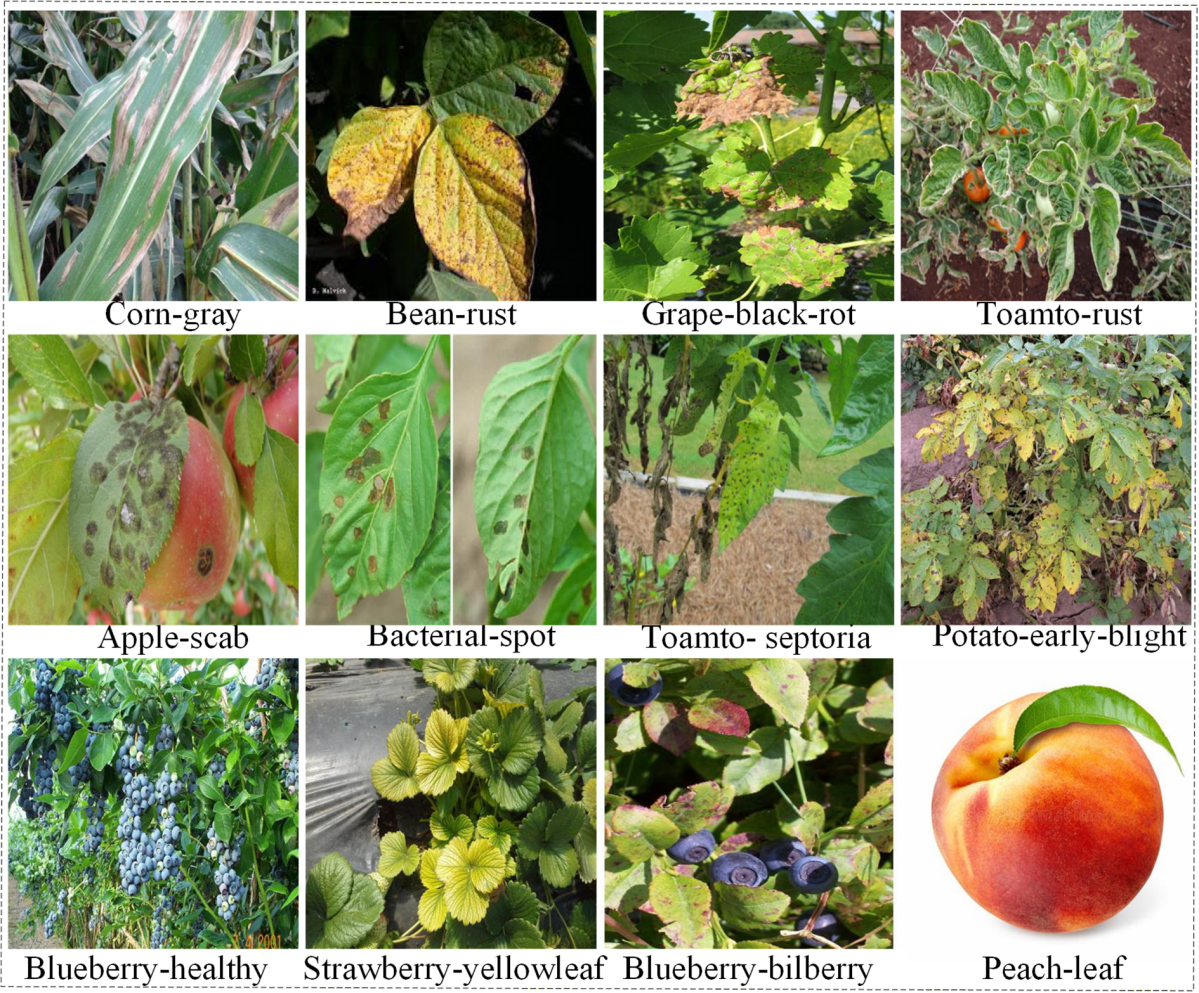
Some samples of PlantDoc dataset.

To further evaluate the generalization capability of the proposed method in agricultural vision tasks, comparative experiments were conducted on the publicly available PlantDoc dataset. PlantDoc is an open dataset for disease detection in real agricultural scenarios, covering multiple crop types and their corresponding disease categories. The dataset exhibits uneven object scale distribution, diverse lesion morphologies, and complex backgrounds, which effectively reflect the practical challenges of object detection tasks in natural cultivation environments. Representative sample images are shown in **Figure 9**.

### 4.2 Experimental environment

All experiments in this study were conducted using the same model parameters and environmental settings. The experimental environment and model parameter configurations are listed in **Table 1**, and the epochs is 150, batch size is 16.

**Table 1:** Experimental environment parameters.

Name	Hardware configuration version	Name	Value
GPU	NVIDIA RTX 4090	Leamingrate (Lr)	0.001
CPU	Intel Core i7	Batch size	16
CUDA	11.8	Optimizer	AdamW
Worker	8	Momentom	0.9
PyTorch	2.6.0	Torchvision	0.21.0

### 4.3 Performance evaluation metrics

In the eggplant fruit pest and disease detection task, multiple evaluation metrics were employed to comprehensively assess the detection accuracy and computational efficiency of the lightweight model in complex natural environments. The metrics include Precision (*P*), Recall (*R*), mean Average Precision (*mAP*@50 and *mAP*@50 − 95), number of parameters (Params), and floating-point operations (*GFLOPs*, True positives (*TP*) are defined as correctly detected fruit borer holes, fruit rot lesions, or thrips feeding traces; false positives (*FP*) occur when fruit surface textures, glare, or other regions are incorrectly identified as pests or diseases; false negatives (*FN*) correspond to missed detections of existing borer holes or lesions, particularly for small-scale targets. The main formulas are presented in Equations 25-28.

(25) $$P=\frac{TP}{TP+FP}$$

(26) $$R=\frac{TP}{TP+FN}$$

(27) $$AP=\int_{0}^{1}P\left(R\right)\;dR$$

Here, *P*(*R*) represents the precision-recall curve with recall as the horizontal axis. The *mAP* is then obtained by averaging over all categories:

(28) $$mAP=\frac{1}{N}\sum_{i=1}^{N}AP_{i}$$

### 4.4 Comparative study

To comprehensively evaluate the performance of various object detection models in the eggplant pest and disease detection task, this study selected mainstream models including YOLOv5n, YOLOv8n, YOLOv10n, YOLOv11n, YOLOv12n, Faster R-CNN, and the RT-DETR r18 variant for experiments on the custom eggplant pest and disease dataset. The comparative experimental results of these models on the publicly available PlantDoc dataset are presented in the **Tables 2**, **3**.

**Table 2:** Comparison of results on the eggplant dataset.

Model	P/%	R/%	mAP@50/%	mAP@50-95/%	Params	GFLOPs	FPS
YOLOv5n	68.1	65.0	70.8	42.1	2.19M	5.8	362.36
YOLOv8n	72.1	67.3	71.7	44.9	2.69M	6.8	328.09
YOLOv9t	73.1	67.7	71.1	46.2	1.77M	6.4	361.91
YOLOv10n	71.2	66.3	69.8	44.1	2.69M	8.2	371.84
Baseline	74.1	69.5	72.7	45.3	2.59M	6.3	325.43
YOLOv12n	75.7	68.6	72.7	45.1	2.50M	5.8	297.67
Faster-RCNN	68.7	42.8	54.0	22.6	41.34M	78.5	91.3
RT-DETR r18	58.1	60.6	65.4	42.7	15.78M	46.0	108.7
DFS-Net (OURS)	**81**.**0**↑	**78**.**3**↑	**80**.**5**↑	**48**.**0**↑	**1**.**8**↓	**5**.**4**↓	**378**.**13**↑

**Table 3:** Comparison of results on the PlantDoc dataset.

Model	P/%	R/%	mAP@50/%	mAP@50-95/%	Params	GFLOPs	FPS
YOLOv5n	50.3	46.2	51.4	38.7	2.5M	7.9	270.27
YOLOv8n	41.3	51.0	49.8	38.7	3.01M	8.1	250.00
YOLOv9t	38.4	50.2	47.5	37.4	1.97M	7.6	232.55
YOLOv10n	48.0	49.6	50.3	39.1	2.27M	6.6	333.33
Baseline	48.4	50.5	52.3	39.0	2.58M	6.3	294.12
YOLOv12n	39.6	48.4	49.6	37.3	2.53M	5.9	256.41
Faster-RCNN	42.5	42.7	51.5	32.3	48.64M	216.31	24.7
RT-DETR r18	38.2	50.7	33.4	33.1	22.75M	62.75	18.3
DFS-Net (OURS)	**52**.**5**↑	**50**.**1**↑	**51**.**8**↑	**39**.**2**↑	**2**.**42**↓	**5**.**9**↓	**319**.**8**↑

Overall, DFS-Net shows stable and competitive performance on both the Eggplant and PlantDoc datasets. On the Eggplant dataset, the proposed method achieves favorable mAP results with fewer parameters and lower computational cost, indicating that the lightweight design contributes to improved efficiency without sacrificing accuracy. On the more challenging PlantDoc dataset, DFS-Net maintains comparable or slightly improved accuracy relative to the baseline, while preserving advantages in inference speed and model compactness. These results suggest that DFS-Net offers a reasonable balance between detection performance and computational efficiency for disease and 517 pest detection in agricultural applications.

### 4.5 Ablation study

To evaluate the contribution of each module to detection performance, YOLOv11n was used as the baseline model, and the PConv, C3K2-MSDA, CSP-MSLA, and SDDH modules were progressively incorporated to ultimately construct the complete DFSNet (OURS) model. The experiments were conducted on the public eggplant pest and disease dataset, and the evaluation metrics described in Section 4.1.2 were employed. The results of the ablation study are presented in **Table 4**.

**Table 4:** Ablation study results of different modules.

Model	PConv	C3K2-MSDA	CSP-MSLA	SDDH	P/%	R/%	mAP@50/%	mAP@50-95/%	Params	GFLOPs	FPS
Baseline	–	–	–	–	74.1	69.5	72.7	45.3	2.59M	6.3	325.43
	✔	–	–	–	78.6	65.3	71.4	44.4	2.54M	6.2	351.51
	–	✔	–	–	76.1	67.8	72.8	45.0	2.56M	6.3	355.86
	–	–	✔	–	79.7	66.6	72.0	44.5	2.54M	6.3	379.56
	–	–	–	✔	82.6	79.0	81.0	48.0	1.8M	5.4	332.00
	✔	✔	–	–	80.0	71.1	74.8	46.8	2.56M	6.25	338.0
	✔	✔	✔	–	81.6	72.3	72.6	47.9	2.58M	6.45	332.0
DFSNet (OURS)	✔	✔	✔	✔	81.0↑	78.3↑	80.5↑	48.0↑	1.8↓	5.4↓	378.13↑

The ablation study results indicate that the contributions of different improvement modules within the network exhibit clear hierarchical and complementary effects. The introduction of PConv in the lower backbone layers stabilizes the preservation of fine-grained features, while the integration of C3K2 and MSDA in the middle and higher layers enhances the network’s capability to represent multi-scale pest and disease targets. On this basis, the incorporation of CSP-MSLA into the Neck stage facilitates more comprehensive feature fusion, improving the information utilization efficiency across different scales. Finally, with the introduction of the SDDH loss function, the model demonstrates better adaptability in target matching and bounding box regression, particularly reflected in improvements in recall and overall detection stability. These results suggest that the modules do not operate in isolation but collaboratively achieve an optimal balance between performance and efficiency.

To further evaluate the effect of each module on the detection performance of different target categories, a category-level comparison of mAP@50 for four detection classes (FruitRot, FruitBorer, Healthy, MelonThrips) was conducted, as presented in **Table 5**.

**Table 5:** Category-level mAP@50 results for different ablation blocks.

Ablation Block	PConv	C3K2-MSDA	CSP-MSLA	SDDH	FruitRot	FruitBorer	Healthy	MelonThrips
	✓	–	–	–	72.8%	60.5%	92.6%	49.7%
	–	✓	–	–	75.4%	63.2%	93.1%	54.6%
	–	–	✓	–	74.6%	62.4%	92.4%	53.8%
	–	–	–	✓	75.1%	63.9%	92.8%	55.2%
	✓	✓	–	–	77.2%	65.1%	93.4%	57.8%
	✓	✓	✓		78.6%	67.0%	93.8%	59.9%
DFS-Net (Ours)	✓	✓	✓	✓	**87**.**3%**↑	**76**.**4%**↑	**94**.**2%**↑	**65**.**1%**↑

The category-level ablation results indicate that the Healthy class exhibits relatively stable performance across different configurations, whereas the FruitRot class shows a consistent improvement trend with the incorporation of multi-scale feature modeling. For small-scale and morphologically complex classes such as FruitBorer and MelonThrips, the collaborative effect of PConv and the multi-scale attention modules significantly enhances feature representation. With the further introduction of the SDDH loss function, the matching and regression performance of these difficult-to-detect classes is improved, leading to overall performance gains that are consistent with the conclusions drawn from the general ablation study.

### 4.6 Visualization

#### 4.6.1 PConv compared to Conv

**Figure 10:**
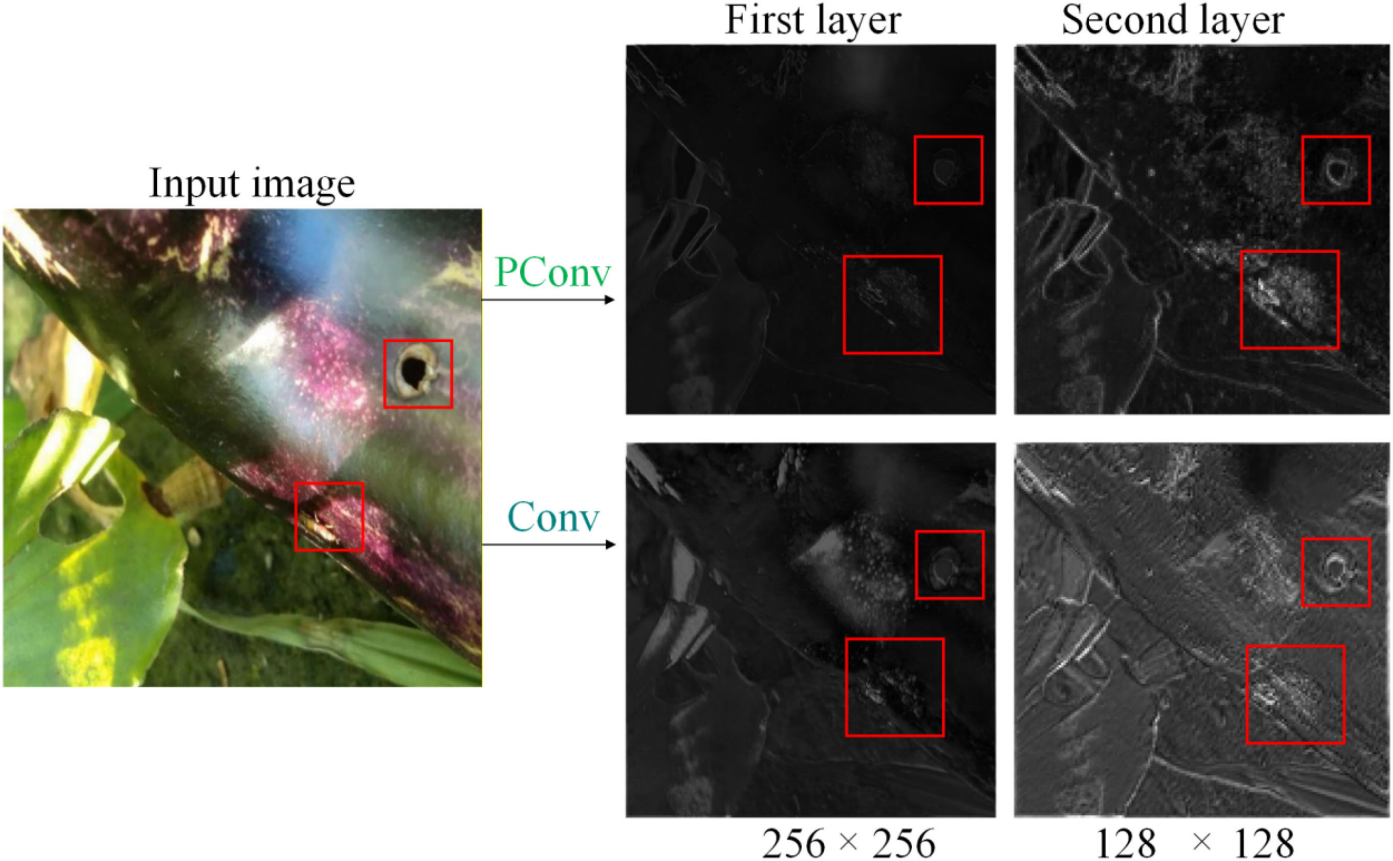
Comparison of shallow feature responses between PConv and standard Conv.

A representative eggplant image containing a FruitBorer hole was selected, and shallow feature extraction at the P1 and P2 layers of the backbone network was performed using both PConv and standard Conv. The comparative results are presented in **Figure 10**. The above comparison indicates that PConv, by employing multi-directional asymmetric convolutional kernels to model local directional structural information in parallel, enables the network to capture more discriminative edge and texture features at shallow layers. This direction-sensitive feature extraction approach facilitates the preservation of critical fine-grained details during subsequent downsampling and multi-scale feature fusion, providing a clear advantage for small-scale targets with prominent edge features, such as FruitBorer holes. In contrast, standard convolution primarily emphasizes the overall local texture distribution at shallow layers, exhibiting limited capability in distinguishing directional structures and fine edges, which may lead to progressive attenuation of small target features under complex natural backgrounds.

#### 4.6.2 Visualization of the model’s feature localization capability

**Figure 11:**
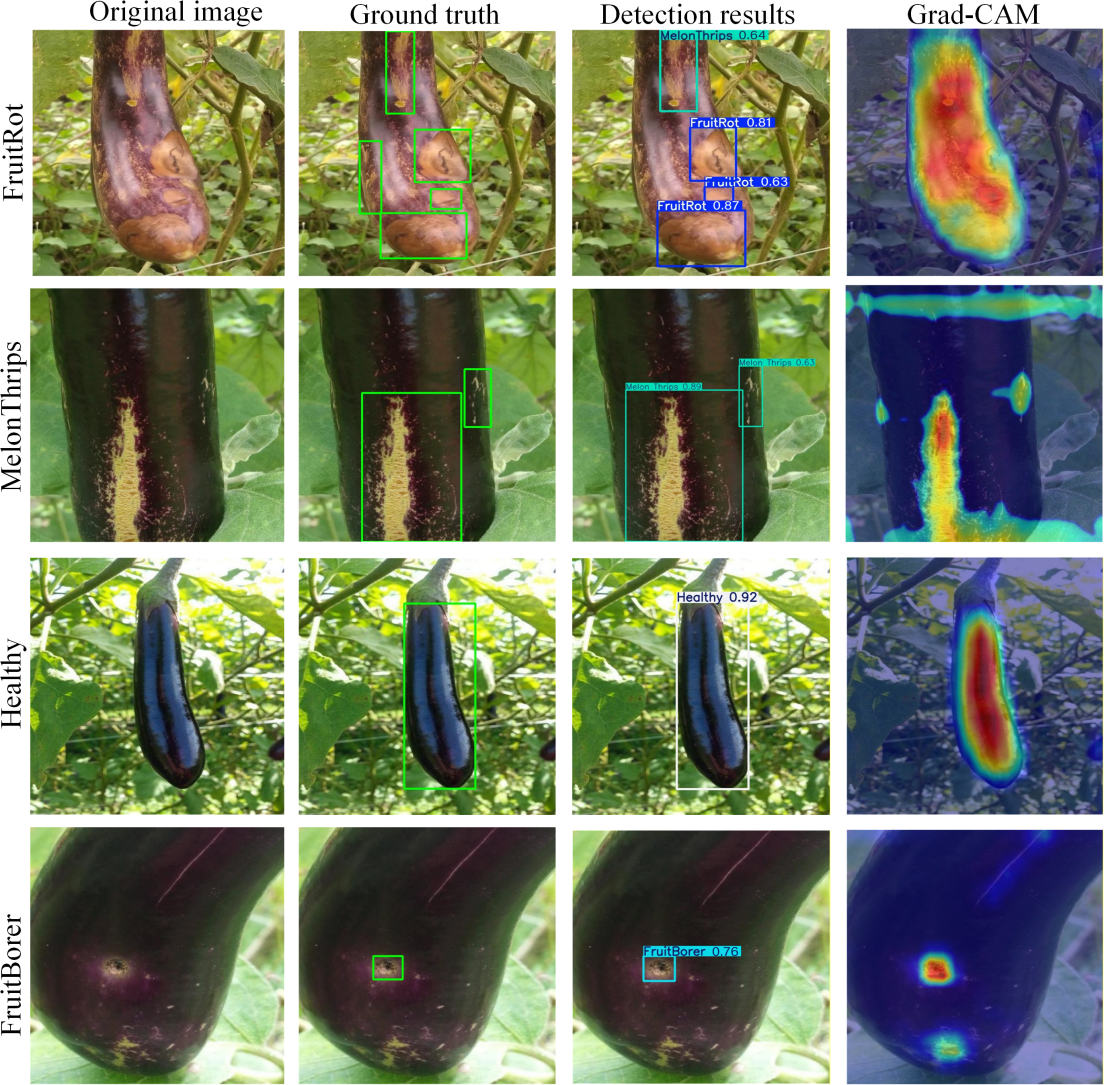
Comparative visualization of detection results and Grad-CAM heatmaps for eggplant fruit diseases and pests.

**Figure 11** presents heatmap comparisons between the proposed DFSNet model and the baseline model on four representative images. These visualizations intuitively reveal the key image regions that the models focus on when detecting different areas or shapes of eggplant fruit diseases and pests. As shown in **Figure 11**, distinct activation patterns can be observed across different categories in the Grad-CAM visualizations. For fruit rot samples, high-response regions are mainly concentrated on the diseased areas and show good spatial consistency with the ground-truth annotations and detection results. For melon thrips, the activation exhibits a clear vertically elongated pattern, which is consistent with the characteristic damage morphology. In healthy samples, no localized abnormal activation is observed, and the responses are primarily distributed over the fruit body. For fruit borer samples, the model produces concentrated activations around the infestation regions. Overall, the heatmap results indicate that the model is able to attend to disease- and pest-related regions while maintaining low responses to background areas.

#### 4.6.3 Qualitative comparison on small fruit rot detection

**Figure 12:**
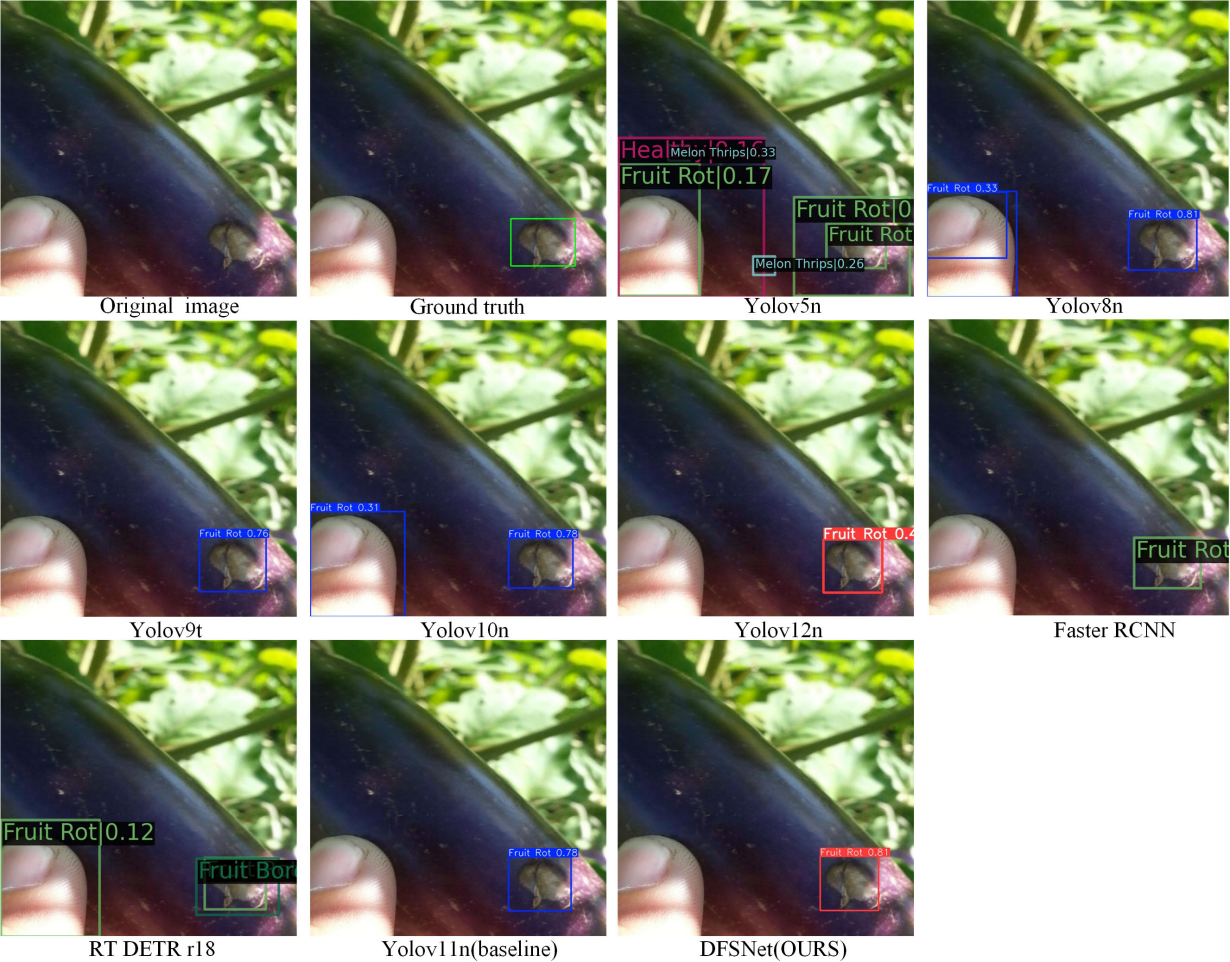
Qualitative detection results.

The **Figure 12** shows DFSNet (OURS) exhibits the best performance on small Fruit Rot detection, providing more accurate localization and higher confidence than other compared models. YOLOv5n–YOLOv12n, Faster R-CNN, and RT-DETR r18 show limited robustness, with low confidence or imprecise bounding boxes under complex backgrounds. These results indicate that DFSNet is more effective in capturing fine-grained features of small disease regions.

#### 4.6.4 Visualization of detection results under complex environments

**Figure 13:**
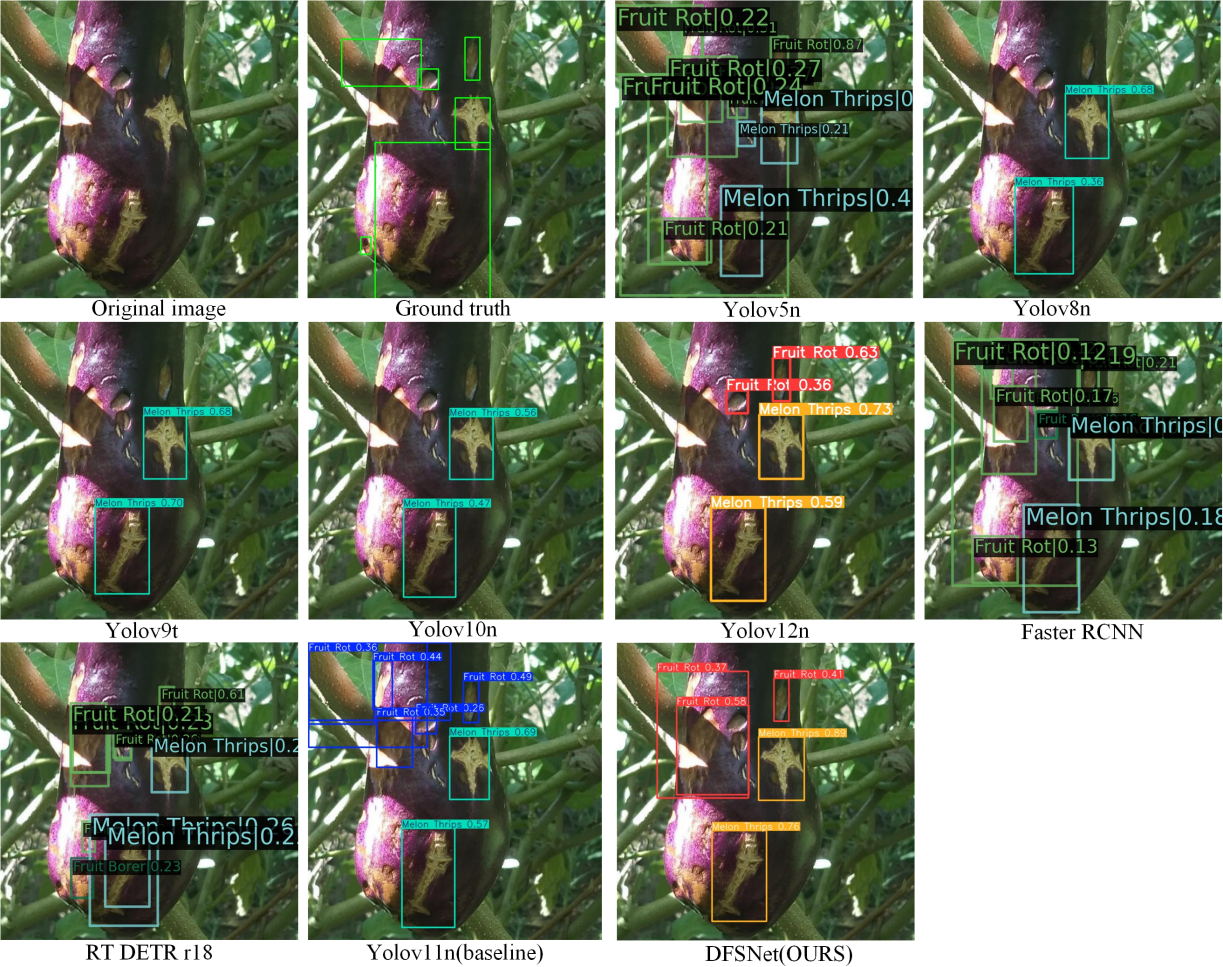
Comparative Performance of Different Models in Complex Environments.

To intuitively compare the performance of different detection models in identifying eggplant fruit diseases and pests under complex greenhouse conditions, representative samples were selected, and the detection results of multiple mainstream object detection models were visualized. As shown in the **Figure 13**, the detection outputs of YOLOv5n, YOLOv8n, YOLOv9t, YOLOv10n, YOLOv12n, Faster R-CNN, RT-DETR-r18, YOLOv11n (Baseline), and the proposed DFSNet are presented for the same scene. Comparative analysis of bounding box positions, class predictions, and confidence distributions allows for an intuitive assessment of each model’s differences in target localization accuracy, class discrimination capability, and adaptability to complex background interference.

The visual comparison of detection results reveals notable differences in the localization and discrimination capabilities of various models under complex backgrounds for eggplant fruit diseases and pests. Some comparative models exhibit overlapping bounding boxes, low confidence scores, or class confusion over fruit surface disease regions, particularly for small-scale targets such as MelonThrips, which are susceptible to occlusion by leaves and interference from background textures. In contrast, DFSNet (OURS) achieves more accurate localization of pest and disease regions, with detection boxes closely aligned with the actual distribution of lesions, while significantly reducing false positives and redundant boxes. Overall, the improved model demonstrates more reliable performance in both target localization stability and class discrimination accuracy, consistent with the results of the previous experiments and ablation analyses, thereby validating the effectiveness and practical applicability of the proposed method in real-world pest and disease detection scenarios.

## 5 Conclusion

This study addresses the task of detecting eggplant fruit diseases and pests under greenhouse conditions, focusing on challenges such as small target scales, diverse morphologies, complex backgrounds, and limited computational resources on edge devices. We propose a lightweight and efficient real-time detection approach. By specifically improving the baseline network architecture, PConv, C3K2-MSDA, and CSP-MSLA modules were incorporated into the backbone and neck structures, and combined with the improved SDDH loss function, resulting in the DFSNet model tailored for complex agricultural scenarios. Experimental results demonstrate that the proposed method achieves a favorable balance between detection accuracy and inference efficiency on the eggplant fruit disease and pest dataset. Compared with various mainstream detection models, DFSNet exhibits superior performance in Precision, Recall, and mAP metrics, while maintaining low parameter counts and computational complexity, satisfying the requirements for real-time detection and deployment in practical natural environments. Ablation studies and visualization analyses further validate the complementary roles of the proposed modules in feature modeling and target discrimination, particularly providing more stable detection for small-scale and morphologically irregular disease and pest targets.

Despite these achievements, there remains room for improvement. First, in scenarios with severe occlusion or significant illumination variations, the model’s recognition of weakly textured lesions could be further enhanced. Second, the current study is primarily validated on a single crop dataset, and the generalization capability of the model across different crops and environmental conditions requires further evaluation.

Future work will explore the integration of more sophisticated cross-scale feature interaction mechanisms or temporal information to enhance model adaptability in complex dynamic scenarios. Additionally, techniques such as knowledge distillation or self-supervised learning could be employed to further improve the detection performance of lightweight models under small-sample conditions. Long-term operational stability and practical deployment of the model on agricultural robots or embedded devices will also be investigated to provide more reliable technical support for intelligent pest and disease monitoring in agriculture.

## Data Availability

The original contributions presented in the study are included in the article/Supplementary Material. Further inquiries can be directed to the corresponding author.
